# Supplementary material for: Label-Free Flow Cytometry: A Powerful Tool to Rapidly and Accurately Assess the Efficacy of Chemical Disinfectants
Source: Microorganisms. 2025 May 19;13(5):1156. doi: 10.3390/microorganisms13051156 (PMC12114458; doi:10.3390/microorganisms13051156)
Supplement: Supplementary file 1 [file microorganisms-13-01156-s001.zip › Supplemental material_Tables S1 and S2.pdf]

**Table S1. Characteristics of bacterial strains used for optimization of the FCM assay.**

| Species | Strain Code | Source | Antibiotic resistance patterns |
|---|---|---|---|
| ***K. pneumoniae*** | Kp1 | unknown | CZ, PRL, AMP, ETR, MEM, IMP, CRO, ATM, CXM, FEP, AMC, FOX, CN, TE, CIP, TOB |
| ***K. pneumoniae*** | Kp2654 | urine | AMP, AMC, TE, CIP |
| ***K. pneumoniae*** | Kp13 | endotracheal tube | CZ, PRL, AMP, ETR, MEM, IMP, CRO, ATM, CXM, FEP, AMC, FOX, CN, TE, CIP, TOB |
| ***K. pneumoniae*** | Kp1427 | unknown | AMP, AMC, TE, TOB, SXT |
| ***K. pneumoniae*** | Kp16 | sputum | CZ, AMP, ETR, MEM, IMP, CRO, CXM, FEP, AMC, FOX, CN, TE, CIP, TOB, SXT |
| ***K. pneumoniae*** | Kp5688 | unknown | CZ, AMP, CRO, ATM, CXM, FEP, AMC, CN, CIP, TOB, SXT |
| ***K. pneumoniae*** | Kp17 | tracheal secretion | CZ, PRL, AMP, ETR, MEM, CRO, ATM, CXM, FEP, AMC, CN, TE, CIP, TOB |
| ***K. pneumoniae*** | Kp2605 | urine | CZ, PRL, AMP, CRO, ATM, CXM, FEP, AMC, CIP |
| ***K. pneumoniae*** | Kp1215 | urine | CZ, PRL, AMP, CRO, ATM, CXM, FEP, AMC, CIP |
| ***K. pneumoniae*** | Kp1982 | tracheal secretion | CZ, PRL, AMP, ETR, MEM, IMP, CRO, ATM, CXM, FEP, AMC, FOX, CIP, AK, SXT |
| ***K. pneumoniae*** | Kp159 | blood | CZ, PRL, AMP, ETR, MEM, IMP, CRO, ATM, CXM, FEP, AMC, FOX, CIP, AK, SXT |
| ***K. pneumoniae*** | Kp164 | urine | CZ, PRL, AMP, ETR, MEM, IMP, CRO, ATM, CXM, FEP, AMC, FOX, CN, TE, CIP, SXT |
| ***K. pneumoniae*** | Kp175 | colonization | CZ, PRL, AMP, ETR, MEM, IMP, CRO, ATM, CXM, FEP, AMC, FOX, CN, TE, CIP, SXT |
| ***K. pneumoniae*** | Kp178 | colonization | CZ, PRL, AMP, ETR, MEM, IMP, CRO, ATM, CXM, FEP, AMC, FOX, CN, CIP, SXT |
| ***K. pneumoniae*** | Kp161 | blood | CZ, PRL, AMP, ETR, MEM, CRO, ATM, CXM, FEP, AMC, FOX, TE, CIP, SXT |
| ***K. pneumoniae*** | Kp23 | urine | CZ, AMP, AMC |
| ***K. pneumoniae*** | Kp19 | varicose ulcer | CZ, AMP, AMC, TE, CIP, SXT |
| ***E. coli*** | Ec2 | urine | AMC |
| ***E. coli*** | Ec13 | urine | AMC, CIP |
| ***E. coli*** | Ec26 | urine | AMC |
| ***E. coli*** | Ec165 | urine | CZ, PRL, AMP, ETR, CRO, ATM, CXM, FEP, AMC, CN, TE, CIP, AK, SXT |
| ***E. coli*** | Ec183 | colonization | CZ, AMP, CRO, CXM, FEP, AMC, TE, CIP, SXT |
| ***E. coli*** | Ec0531 | urine | CZ, AMP, CRO, ATM, CXM, FEP, AMC, TE |
| ***E. coli*** | Ec17 | urine | CZ, PRL, AMP, CTX, ATM, CXM, FEP, AMC, TE, CIP, SXT |
| ***E. coli*** | Ec18 | urine | CZ, PRL, AMP, CTX, ATM, CXM, FEP, AMC, CN, TE, CIP, SXT |
| ***E. coli*** | **Ec10538** | **ATTC reference strain** | - |
| ***A. baumannii*** | Ab20 | bronchial secretion | SAM, IMP, MEM, DOR, FEP, CN, AK, CIP, CAZ |
| ***A. baumannii*** | Ab110 | tracheobronchial aspirate | SAM, IMP, MEM, DOR, FEP, ATM, CN, AK, CIP, CAZ |
| ***A. baumannii*** | Ab111 | sputum | SAM, IMP, MEM, DOR, FEP, ATM, CN, AK, CIP, CAZ |
| ***A. baumannii*** | Ab88 | sputum | SAM, IMP, MEM, DOR, FEP, ATM, CN, AK, CIP, CAZ |
| ***A. baumannii*** | Ab108 | skin infection | IMP, MEM, DOR, FEP, CN, AK, CIP, CAZ |
| ***P. aeruginosa*** | Ps1696 | secretion | TZP, MEM, IMP, FEP, ATM, CAZ, AK, DOR, CN, CIP, TOB |
| ***P. aeruginosa*** | Ps1707 | tracheal secretion | CAZ, ATM, FEP, MEM, IMP, AK, TOB, CIP, CN, DOR |
| ***P. aeruginosa*** | Ps1689 | secretion | IMP, ATM, CAZ, FEP, MEM, AK, DOR, CN, TOB, CIP |
| ***P. aeruginosa*** | **Ps15442** | **ATCC reference strain** | - |
| ***S. aureus*** | Sa13358 | hemoculture | FOX, AZM, P, E, TE |
| ***S. aureus*** | Sa47 | Skin infection | FOX, AZM, P, CN, E, TE, LZD |
| ***S. aureus*** | Sa25 | unknown | P |
| ***S. aureus*** | Sa112 | unknown | AZM, P, CN, CIP, E |
| ***S. aureus*** | Sa10 | Skin infection | FOX, AZM, P, E, TE |
| ***S. aureus*** | Sa110 | Skin infection | P |
| ***S. aureus*** | **Sa6538** | **ATCC reference strain** | - |
| ***E. faecium*** | Ef15 | tracheal secretion | TE, CN, CIP, P, VA |
| ***E. faecium*** | Ef16 | tracheal secretion | TE, CN, CIP, P, VA |
| ***E. faecium*** | Ef19 | tracheal secretion | TE, CN, CIP, P, VA |
| ***E. hirae*** | **Eh10541** | **ATCC reference strain** | - |

Legend: SAM Ampicillin/Sulbactam, CZ Cephazolin, AMC Amoxicillin-clavulanic acid, FOX Cefoxitin, AZM Azithromycin, P penicillin, TE tetracycline, CN gentamycin, E erythromycin, CIP ciprofloxacin, ATM aztreonam, DOR doripenem, AK amikacin, FEP cefepime, PRL piperacillin, MEM meropenem, IMP imipenem, LZD linezolid, CXM cefuroxime, CAZ ceftazidime, CRO ceftriaxone.

Table S2 Comparative Performance of the FCM Method and Standard Suspension Tests for Disinfectant Efficacy Assessment.

| **Disinfectants** | **False positive results** | **False negative results** |
|---|---|---|
| Mikrozid™ | 0 | 2 |
| Klinosept™ | 0 | 3 |
| Glutanol™ | 0 | 0 |
| Peroklin™ | 0 | 1 |
| Clor2Klin™ | 0 | 0 |
